# Lipid metabolism within the bone micro-environment is closely associated with bone metabolism in physiological and pathophysiological stages

**DOI:** 10.1186/s12944-021-01615-5

**Published:** 2022-01-07

**Authors:** Bo Wang, Heng Wang, Yuancheng Li, Lei Song

**Affiliations:** 1 Department of Orthopedics, The first affiliated Hospital of Army Medical University, Army Medical University, Gaotanyan Street No. 30, Chongqing, 400038 China; 2 Battalion of Basic Medical Sciences, Army Medical University, Chongqing, 400038 China

**Keywords:** Lipid metabolism, Bone metabolism, Bone destruction diseases

## Abstract

Recent advances in society have resulted in the emergence of both hyperlipidemia and obesity as life-threatening conditions in people with implications for various types of diseases, such as cardiovascular diseases and cancer. This is further complicated by a global rise in the aging population, especially menopausal women, who mostly suffer from overweight and bone loss simultaneously. Interestingly, clinical observations in these women suggest that osteoarthritis may be linked to a higher body mass index (BMI), which has led many to believe that there may be some degree of bone dysfunction associated with conditions such as obesity. It is also common practice in many outpatient settings to encourage patients to control their BMI and lose weight in an attempt to mitigate mechanical stress and thus reduce bone pain and joint dysfunction. Together, studies show that bone is not only a mechanical organ but also a critical component of metabolism, and various endocrine functions, such as calcium metabolism. Numerous studies have demonstrated a relationship between metabolic dysfunction in bone and abnormal lipid metabolism. Previous studies have also regarded obesity as a metabolic disorder. However, the relationship between lipid metabolism and bone metabolism has not been fully elucidated. In this narrative review, the data describing the close relationship between bone and lipid metabolism was summarized and the impact on both the normal physiology and pathophysiology of these tissues was discussed at both the molecular and cellular levels.

## Introduction

Both cholesterol and lipid metabolites are widely distributed throughout the human body and play a vital role in several metabolic pathways. Cholesterol, which is primarily produced in the liver, is commonly found throughout the human body. Cholesterol synthesis is a tightly regulated process facilitated by several key enzymes, including 3-hydroxy-3-methylglutaryl-CoA (HMG-CoA) reductase, which is a key target for the regulation of these pathways. There has been a recent increase in the number of studies designed to evaluate the relationship between lipids and bone metabolism [1, 2]. This is likely because changes in both lipid and cholesterol metabolism can result in significant disruptions in the bone microenvironment. Previous studies have shown that individuals with increased body mass are more likely to experience bone fractures, which can be partly explained by the effects of gravity, leaving a large question about the impact of metabolic disorders on bone strength. It is likely that disruptions in lipid metabolism may result in changes in normal bone homeostasis and an increase in osteopathies, such as osteoarthritis and fractures.

## Intercellular crosstalk during bone metabolism

Bone is usually found in a single constant state, with a normalized structure and function. The interrelationship between osteoblasts and osteoclasts contributes to a clear coupling between bone absorption and remodeling, allowing for a balance between bone loss and regeneration. There are several ways in which osteoblasts come into contact with osteoclasts, including cell-cell surface interplay, cytokine or paracrine signaling, and extracellular matrix contact. Studies have shown that osteoblasts and osteoclasts interact with each other through a variety of signaling pathways [3, 4].

Cell-cell communication between osteoclasts and osteoblasts during bone remodeling can be divided into four states: Initially, osteoclasts recognize the injury site and the resultant calcium concentrate and then undergo cell fusion, polarization, and shape change, producing a clear zone around the injury site, which is then bordered by a subset of osteoclasts that bind tightly to the bone surface in order to seal the area from unwanted interactions. This process is referred to as the activation state. This coincides with the establishment of the ruffled border, which is designed to produce hydrogen cations (H^+^) and enzymes needed to resolve the bone matrix. The second phase is the absorption state, in which several enzymes are released into the microenvironment, including cathepsin K and matrix metalloproteinases (MMPs). This leads to the transverse state, where the hematopoietic stem cells (HSCs) differentiate into osteoblasts, inducing the production of various cytokines from osteoclasts, including insulin like growth factor (IGF-1) and transforming growth factor (TGF-β), to produce critical chemotactic gradients used to recruit osteoblasts to the bone remodeling site. Osteoclasts also produce osteoprotegerin (OPG), inhibiting receptor activator of NF-kB (RANK) function, facilitating proper metabolic balance [5], and acting as a stop signal for bone absorption. The transverse state plays a key role in many bone diseases. This is facilitated by osteoclasts continuing the breakdown of the bone matrix and facilitating bone absorption when the transition state fails. The bone debris produced during absorption affects this state. For example, the denatured matrix induces T cell-mediated receptor activator of NF-kB ligand (RANKL) production via dendritic cell activation, which continues bone destruction and supports osteoclast survival. The last stage in bone remodeling is to produce new bone at the injury site. Here, the osteoblasts participate in matrix indisposition and osteocyte mineralization.

Studies have shown that osteoblasts form gap junctions with osteoclasts, facilitating ion transactions [6]. These gap junctions are facilitated by EphB4 on osteoblasts and Ephrin2 on osteoclasts and enables the osteoblast-osteoclast balance in these environments. Osteoblasts may regulate osteoclast differentiation by creating a cell niche [7].

On the molecular level, macrophage colony-stimulating factor (M-CSF), which is essential for osteoclast differentiation, is commonly produced by osteoblasts [8] and secrete lysophosphatidic acid (LPA), which negatively affects osteoclast development [9]. Under inflammatory conditions, osteoblasts are stimulated to produce Monocyte chemoattractant protein-1 (MCP-1) to recur preosteoclasts and cope with M-CSF to mature osteoclasts. The OPG/RANK/RANKL balance also facilitates communication between these cells [10] as osteoblasts secrete soluble RANKL when activated by vitamin D_3_ or the parathyroid hormone (PTH), which in turn induces osteoclast activity. However, these cells also produce OPG, which inhibits the activity and proliferation of osteoclasts [10]. Additionally, RANKL derived from osteocytes in mineralized bone matrices makes sense in bone destruction [11]. Membrane-bound RANKL, a member of the tumor necrosis factor (TNF) superfamily [12], facilitates cell-cell communication between osteoblasts and osteoclasts. RANK is often found on the membrane of osteoclasts. Following osteoclast-mediated bone absorption, debris from the bone matrix interacts with dendritic cells, which in turn induces CD4^+^naive T cells to produce RANKL and display this on their cell surface, while adipose cells produce OPG, which counterbalances RANKL expression changes. In addition, when the bone experiences inflammation, the collar osteoblasts up-regulate MCP-1, which makes it easier for osteoclasts to recognize and attach to each other [13]. Osteoclast-derived sclerostin and semaphorin 4D not only inhibited osteoblast differentiation but also downregulated osteoblast production of RANKL.

**Fig. 1:**
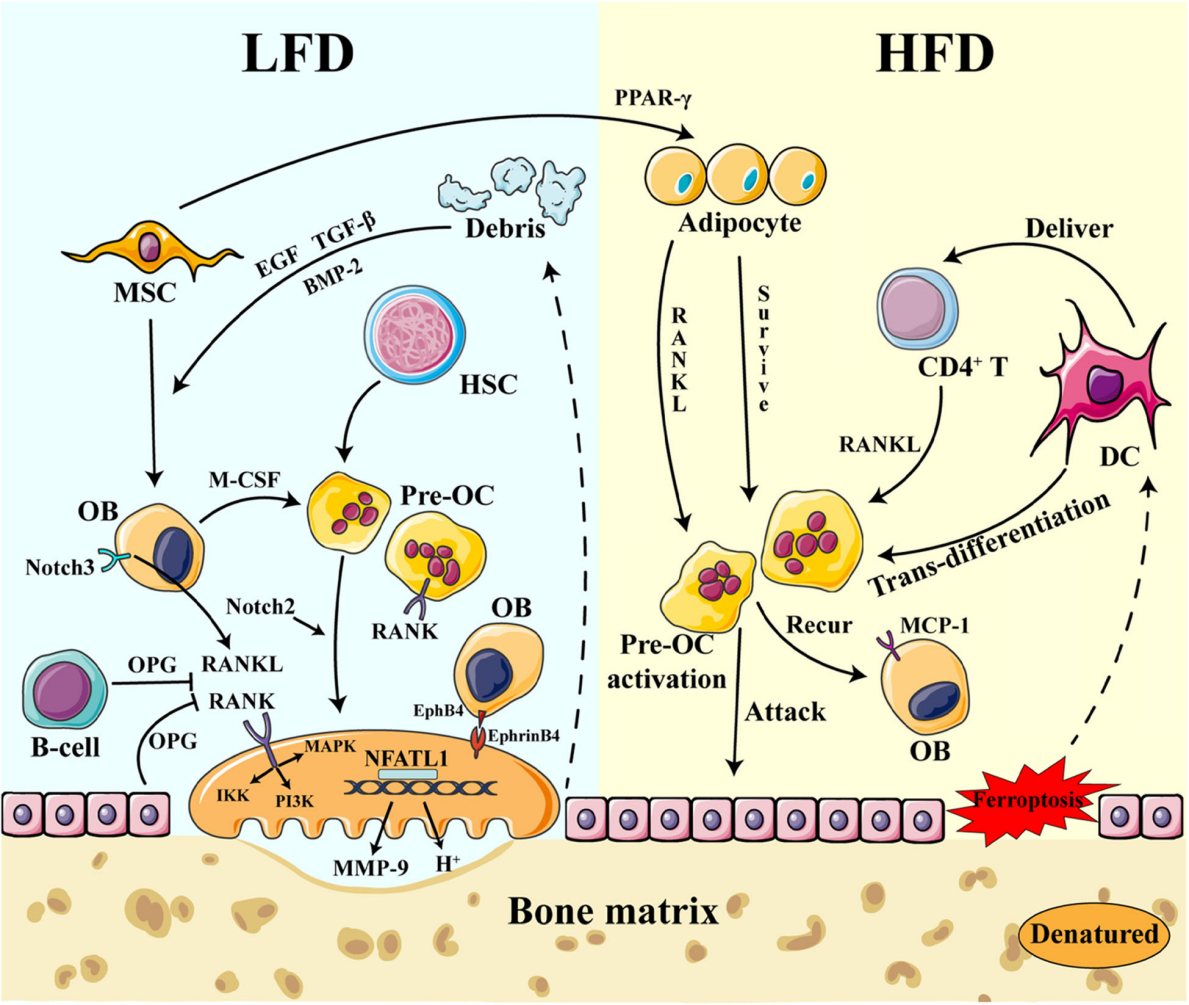
The activation and function of several cell types in a low fat diet (LFD) and high fat diet (HFD micro-environment. In a LFD and low cholesterol micro-environment, MSCs were prone to differentiate into osteoblasts with low pre-osteoclast formation and molecular and cellar signal and a high osteoblastic signal. In this state, due to low adipose derived cytokines, osteoblasts and osteoclasts were toned well with several cells taken part in, such as collar cell and B cells. However, in a high fatty diet, MSCs in the cell niche were exposed to a high fatty signal, making them more likely to differentiate to adipocytes. Adipocytes then become another vital source of RANKL, a molecule that is necessary for osteoclasts formation and function. Simultaneously, HFD and high cholesterol makes it more likely for the collar osteoblasts to undergo ferroptosis. The denatured matrix is prone to attract dendritic cells, whose activation could produce RANKL for osteoclasts in the pathology state. While RANK is activated, several pathways are involved to activate NFATc1 to fulfill bone absorption

At the cellular signaling level, interactions between osteoblasts and osteoclasts activate the RANK-RANKL signaling pathway, which regulates the production of TNF-receptor associated factor 6 (TRAF6), and the activation of classic NF-kB signaling, mitogen-activated protein kinase (MAPK), and c-fos, which are all linked to the regulation of bone absorption and the production of MMPs, tartrate resistant acid phosphatase (TRAP), and cathepsin K [14]. Furthermore, the nuclear receptor and transcription factor, peroxisome proliferators-activated receptor (PPAR-γ), is likely to participate in OB-OC interactions, making it a critical target for therapeutic intervention (Fig. 1).

However, more studies are focusing on adipose cell-derived cell factors in osteoblast-osteoclast communication. Leptin induces the differentiation of MSCs into osteoblasts, while inhibiting their differentiation into adipose cells. In addition, studies have shown that several molecules are involved in the osteoblast-osteoclast crosstalk in this stage, such as apolipoprotein E (APOE) and adipokines [15, 16]. However, leptin may experience positional specificity in weight-bearing and non-weight-bearing bones. High lipid and cholesterol concentrations also increase bone cell exposure to the inflammatory microenvironment: First, high fatty acid and cholesterol levels increase the possibility of oxidation of unsaturated fats, which are more likely to become the target of reactive oxygen substrate (ROS) or poly unsaturated fatty acid radicals (PUFA-OO^**.**^). High fat environments also produce more ‘fatty factors’ which in turn produce a more inflammatory phenotype resulting in the reversion of bone cells to preosteoclasts stage leading to changes in differentiation, maturation and survival. Simultaneously, these factors result in the dysfunction of the osteoblastic differentiation of MSCs and induce increased adipogenic activity.

## The role of cholesterol and the lipid metabolism in bone micro-environment

Bone remodeling requires a steady balance between bone formation and destruction throughout life. The counterbalance between osteoblasts and osteoclasts plays a vital role in regulating the cell cycle. Studies have shown that lipids and cholesterol will make sense in their communication at each stage.

### Cross talk between osteoblasts and cholesterol contribute to bone homeostasis

Bone homeostasis depends on several different cells and factors, with osteocytes, osteoblasts, and osteoclasts being the most important. Osteoclasts are derived from hematopoietic stem cells and share a common precursor with macrophages. Osteoblasts are differentiated from mesenchymal stem cells (MSCs), which also possess the ability to differentiate into adipose and chondrogenic cells. These cells also participate in fibrocyte, chondrocyte, and myoblast production, but the primary differentiation balance is centered around adipose cells and osteoblasts [17]. Despite the self-renewal capacity of these stem cells, when MSC differentiation favors one cell type over another there is less of the other cell type, i.e. the more adipose cells produced in a system the fewer osteoblasts there will be. This is because adipogenic factors, such as PPAR-γ, exert a strong inhibitory effect on the production of osteogenic signals [18]. Other adipogenic molecules, including adiponectin or charerin, also contribute to the differentiation balance [19]. These forces counteract each other under normal physiological conditions and maintain cellular balance. In addition, extracellular factors from adipose cells can affect osteoblastsogenic, such as leptin [20], but the concrete significance of leptin remains to be understood [21, 22].

Recent studies have revealed a relationship between osteoblasts and cholesterol metabolism. Notch, a key biomarker for osteoblasts, contributes to many of the signaling interactions required for osteoblast differentiation, formation, and function [23, 24]. The Hey family, including Hey1, Hey2, and HeyL, are Notch targets that function as support during bone absorption. Recent studies have revealed that lipid metabolism can alter mid-gut stem cell differentiation via Notch signaling in Drosophila, demonstrating a relationship between Notch, gastrointestinal development, and cholesterol [25]. The stability of the Notch protein outside the cell membrane is regulated via lipid control, especially cholesterol [26]. Wnt-βcatenin is also likely to participate in the relationship between osteoblasts and cholesterol [27]. Recent evidence has shown that cholesterol modulates Xenopus development via the Wnt–β-catenin pathway, while Wnt-βcatenin plays important role in embryonic skeletal development and regulating articular cartilage, synovium, and osteoblasts in adults [28]. The proper orchestration of both these signaling pathways with lipids and cholesterol is critical for bone production.

### Changes in osteoclasts in response to metabolic dysfunction

When collagen is denatured, for instance in rheumatoid arthritis (RA) [29], MSCs are more likely to differentiate into adipose cells. Postmenopausal women are more likely to experience osteoporosis because of changes in the bone marrow MSC (mMSC) differentiation balance, which tips towards adipose cells and away from osteoblast differentiation following these hormonal changes [30]. The mMSCs of these women exert a reduced protective effect against bone loss and they are more likely to experience bone fractures resulting from the increased number of adipose cells inside the bone matrix.

Cholesterol interacts with osteoclasts in various ways, including its dysregulation of osteoclast differentiation at high concentrations. This is likely due to the increase in cholesterol content within the bone marrow that reduces MSC differentiation into osteoblasts by increasing adipogenic signaling, which overwhelms osteogenic signalling [31]. While more osteoclasts are activated, there is less marrow space [31], enabling cholesterol to fill the space and activate even more osteoclasts. Simultaneously, high cholesterol is essential for osteoclast survival [32–34]. For example, these osteoclasts undergo polarization, and cholesterol and fatty acids make their cell membranes more flexible [33]. When the cholesterol inside the osteoclasts is reduced or depleted, it will induce osteoclast apoptosis [35]. In addition, adipose cell-derived factors are essential for osteoclast development and maturation. Adipose cells may act as an additional source of RANKL [36–38], activating osteoclasts and tipping the metabolic balance in the osteocyte niche.

Studies predict that, like most monocytes, macrophages express a scavenger receptor that collects lipoproteins, similar to those in osteoclasts. Osteoclasts are also differentiated from monocytes, so it is easy to understand why they have the ability to absorb cholesterol. In other rat models, a high cholesterol diet (HCD) reduced the density of alveolar bones and the number of tartrate-resistant acid phosphatase (TRAP)-positive osteoclasts increased significantly [39]. This bone destruction can be ameliorated by adding vitamin C and provides strong evidence that oxidative stress is induced by HCD, which can be sensed by osteoclasts [40]. Cholesterol acts as the primary source of the ROS. Recent studies have shown that iron-induced lipid peroxide may cause membrane lipid dysfunction and induce the production of poly-unsaturated fatty acid peroxide substrates and cell death (ferroptosis) [41, 42]. Ferrous elements promote ROS production via Fenton reactions with hydrogen peroxide. ROS thereafter attacks the cell membrane and induces cell lysis and death. This is exacerbated by aging, where bone mass declines and lipid and cholesterol concentrations increase, especially in postmenopausal women [43]. When the bone marrow is saturated with lipids and cholesterol, it is more likely to experience an increase in ferroptosis. At the same time, stem cells in this niche will experience increased ROS stress, further reducing their capacity to migrate and repair the niche or regulate bone remodeling. This results in failure to differentiate into osteoblasts, widening cell type imbalance, and pushing the aberrant differentiation of these cells. Moreover, Oxidation oxidized low density lipoprotein (ox-LDL) increases under these conditions and has further implications [44]. In summary, these results suggest that with cholesterol and unsaturated fatty acids filling the bone marrow, there is a sharp increase in ROS production following the destruction of the phospholipid bilayer. This affects bone remodeling by enhancing osteoclast function while inhibiting osteoblast and MSC repair. Recent studies have shown that (co-enzyme Q reduction state) CoQH_2_ and ferroptosis suppressing protein (FSP1) function as effective inhibitors of ferroptosis in tumor models. FSP1 seems to bond the lipid membrane structure, especially the cell membrane and mitochondrial membrane; however, the effects of inhibiting ferroptosis in the bone structure remain unclear [45]. This might be the next hot issue to discuss. Interestingly, CoQs are associated with the mevalonic acid (MVA) pathway, which is essential for cholesterol production. This means that cholesterol metabolism and its linked metabolic pathways probably play a double-edged role in bone homeostasis; simultaneously inducing bone destruction whilst having a protective role. Furthermore, low density lipoprotein (LDL) influences osteoclast function. High fat diet (HFD) induced increase in LDL make it more difficult to overcome osteoclast signaling during the transverse state [32, 46, 47]. However, studies have shown that there is a more complex interaction in these cells, dependent on both LDL and ox-LDL. High LDL levels inhibit the number of osteoclasts, which results in a co-stimulatory effect on the inflammatory pathways associated with joint disease. In contrast, ox-LDL inhibits osteoclast function and differentiation [48, 49].

## The relationship between cholesterol metabolism and bone diseases

### Cholesterol metabolism and osteoporosis (OP)

OP is a systemic bone disease characterized by decreased bone mass and deterioration of the bone tissue microstructure, resulting in increased bone brittleness and fracture [50]. OPs are generally divided into two types. The first is primary or age-related OP, which has no obvious cause, and its incidence is higher in women than in men, especially in older people. OP is particularly prevalent in postmenopausal women due to bone loss associated with decreased estrogen levels, demonstrating a clear disposition toward increased fractures. Secondary OP refers to bone loss caused by other diseases, which affects both men and women and can be associated with a variety of metabolic diseases, including rheumatoid arthritis, hyperparathyroidism, Cushing’s disease, and chronic kidney disease. Secondary OP can also be induced by certain drugs such as anti-epileptic drugs, glucocorticoids, and lithium, as well as eating disorders, cancer, and organ transplants.

As an increasing number of studies have focused on these relationships, the link between cholesterol metabolism and OP during lipid metabolism has become clearer. Cholesterol metabolism plays an important role in various types of OP. Previous studies have shown that hypercholesterolemia is closely related to osteoporosis, and there is a negative correlation between bone mass and total cholesterol (TC) levels in postmenopausal women [51]. An OP mouse model demonstrated that glucocorticoid stimulation resulted in adipocyte aggregation, increased cholesterol levels, and decreased bone mineral density (BMD) [52]. This experimental evidence suggests that changes in serum cholesterol levels and their related lipoproteins damage the intrinsic balance in bone metabolism, resulting in bone loss. Given this, it is unsurprising that a large number of recent studies have focused on the underlying mechanisms of these interactions. These results have revealed that cholesterol-mediated changes in bone metabolism are linked to one of two aspects: its effects on bone homeostasis. High cholesterol diets significantly decrease bone mineral density and the serum concentration of osteogenic markers, while increasing the level of bone resorption markers in these samples [53]. Additionally, other studies have shown that HCD inhibit the proliferation and differentiation of mouse osteoblast MC3T3-E cells in a dose-dependent manner [53]. After cholesterol treatment, the expression of osteogenic genes such as alkaline phosphatase (ALP, ALPL), collagen type 1 (COL2A1), bone morphogenetic protein (BMP2), and dwarf related transcription factor 2 (Runx2) decreased, and the normal expression of these genes is an important factor in the osteogenic process. This suggests that free cholesterol might block the expression of Runx2, ALPL, and Col1a1 in osteoblasts by inhibiting BMP2, thereby inhibiting the differentiation of these osteoblasts. In addition, gene map analysis showed that high-cholesterol diets may inhibit TGF-β /BMP2/Wnt signaling [53]. TGF-β / BMP2 signaling is of critical importance for bone production in mammals, while Wnt signaling is responsible for almost all osteoblast functions [54, 55]. Both the bone content and density of the femur in mice fed a high cholesterol diet were significantly lower than those in mice fed a simple diet [56]. In addition to its effect on osteogenesis, cholesterol plays an important role in the process of bone fracture. Pelton et al. found that the number of osteoclasts in the hypercholesterolemia group significantly increased when compared to the control group, resulting in a decrease in bone mineral density, bone volume fraction, and the number of bone trabeculae, and an increase in trabecular spacing, and the concentration of circulating osteoclast markers, namely type I collagen pyridinoline cross-linked fragments [57]. A high-cholesterol diet increased the number of tartrate-resistant acid phosphatase (TRAP+)-resistant osteoclasts, resulting in a decrease in alveolar bone mineral density. These changes were partially restored following treatment with vitamin C [58]. Vitamin C acts as a reducer, reducing oxidative damage, which in turn promotes osteoblast differentiation and increases type I collagen production. This suggests that anti-oxidative stress drugs may help to resist osteoclast activation in response to hypercholesterolemia and protect the bone [59]. LDL is also an important carrier of serum cholesterol, and has been shown to promote osteoclast production, because osteoclasts have only a very low expression level of HMG-CoA reductase, a key enzyme in cholesterol [60], and its expression level is not upregulated when cholesterol is depleted on the cell membrane [60]. This means that exogenous cholesterol is more important for osteoclasts, highlighting the importance of cholesterol carrier LDL. LDL activates osteoclasts and promotes bone destruction by efficiently delivering cholesterol to these cells. This is further validated by the fact that low-density lipoprotein receptor-negative (LDLR-) mice experienced an increase in bone mass [34], a decrease in bone resorption, and reduced osteoclast number, size, and lifespan. These animals also exhibited more spontaneous apoptosis and delayed osteoclast differentiation, with fewer nuclei. However, there was no significant change in bone production, indicating that exogenous LDL plays an important role in osteoclast formation, while LDLR deficiency inhibits osteoclast formation and fusion [61]. In contrast, disorders in cholesterol metabolism have a negative regulatory effect on bone microcirculation, which is closely related to the development of OP. Disturbances in cholesterol metabolism may lead to dysfunction of the intraosseous vascular endothelial cells, decreased nitric oxide secretion, and increased endothelin production, increasing the risk of thrombosis [62]. High doses of corticosteroids promote cholesterol synthesis, increasing the risk of fat deposition, liver steatosis, and fat embolism [63]. At the same time, cholesterol metabolism dysfunction promotes the development of adipocytes in the medullary cavity, which increases the average diameter of adipocytes in the bone marrow by 10 μm [64], which increases medullary cavity pressure and affects perfusion by activating the blood coagulation pathway [65, 66]. Increases in circulating cholesterol levels lead to lipid accumulation in the bone marrow, and the development of accompanying fat emboli, which can result in subchondral vascular occlusion, reductions in cartilage and bone blood perfusion, and increases in the occurrence and development of OP. Several studies, such as those by Broulik and Tang [67, 68], have confirmed that cholesterol metabolism disorders can have a negative effect on bone status. If this is true, one must ask if the reduction of cholesterol can protect the bones. The answer is not that simple. Cholesterol is a necessary component of animal cell membranes, and a number of experiments have confirmed that the effects of cholesterol on bone tissues is not simply “good” or “bad.” Pharhami et al. [69] proved that osteogenic differentiation of MSCs requires a baseline level of cholesterol synthesis, and that the osteogenic differentiation capacity of MSCs was severely impaired when there was insufficient cholesterol available. In addition, increases in triglyceride (TG), low-density lipoprotein cholesterol (LDL-C), and apolipoprotein B (ApoB) levels and decreases in high-density lipoprotein cholesterol (HDL-C) and apolipoprotein A (ApoA) are beneficial to bone metabolism. This conclusion is less widely accepted in studies by Samelson EJ [70] and Tank et al. [71]. Li et al. [72] confirmed the effect of cholesterol on bone, especially its effects on the osteogenic process, and showed that these outcomes are closely associated with the source of cholesterol. Exogenous cholesterol inhibits osteoblast differentiation, while physiological levels of endogenous cholesterol are necessary for osteogenic differentiation of bone marrow stem cells. This is consistent with the conclusions of Pharhami [69]. Further studies confirmed that under the condition of maintaining the normal expression of Runx2, Col1a1, and osteocalcin (Bgalp), targeted inhibition of cholesterol biosynthesis pathway, alkaline phosphatase (ALP) activity expression, and osteoblast mineralization decreased synchronously, suggesting that endogenous cholesterol is necessary for osteogenic differentiation [69]. These results show that cholesterol not only damages the bone, but normal physiological levels of cholesterol are critical for the maintenance of normal bone metabolism. Yamaguchi et al. [73] investigated the correlation between blood lipid levels and BMD in postmenopausal Japanese women and analyzed the relationship between serum LDL-C levels and spinal fractures. They found that serum LDL-C levels were negatively correlated with BMD. Low high-density lipoprotein (HDL) levels were also found to be associated with an increased risk of fracture. Other experiments have also confirmed that patients with postmenopausal osteoporosis have higher levels of serum LDL and TC [74]. In addition, estrogen supplementation can treat postmenopausal osteoporosis by reducing the levels of TC and LDL-C [51, 75]. Another group of observational experiments concluded that changes in the serum cholesterol indicators were not unique to postmenopausal women, but could also be identified in women of any age when they developed osteoporosis. Broulik et al. matched 241 Czech women with osteoporosis to 98 women of similar age and found that osteoporotic women with spinal fractures had significantly higher cholesterol levels than their peers [67]. In addition, patients with type 2 diabetes mellitus (T2DM) and OP presented with elevated levels of TC, TG, and LDL-C when compared to those without OP, while the level of HDL-C was significantly lower than that in patients without OP [76]. A study by Sivas et al. [77] showed that TC levels were the largest factor affecting the risk of persistent spinal fracture, with each increase of 1 mg/dL in TC level, reducing the risk of vertebral fractures by 2.2% (paired 0.009). Other studies, such as those completed by Dennison et al. [78], have shown that fasting HDL-C levels in women are associated with changes in lumbar bone mineral density. There was a negative correlation between the HDL-C/LDL-C ratio and total bone mineral density in the femurs of both male and female participants. In addition, the total bone mineral density of the spine was negatively correlated with the level of ApoA but positively correlated with the level of ApoB [78].

Statins are primarily used to treat hypercholesterolemia, as they can reduce serum cholesterol by competitively inhibiting HMG-CoA reductase, a key rate-limiting enzyme in the cholesterol biosynthesis pathway [79]. A number of clinical and animal experiments have shown that statins can promote bone production, inhibit osteolytic metastasis, reduce the risk of fracture, and play a significant role in bone protection [71, 80–82]. This effect is closely related to drug concentration [83]. The osteoprotective effects were initially discovered by accident when identified in murine screens for BMP2 enhancers [84]. Mundy et al. [84] injected lovastatin and simvastatin into the skulls of mice and found that these injections resulted in significant bone mass increases in the skulls of these mice, suggesting that they can promote osteogenesis. Statins can be divided into two groups: relatively fat-soluble (such as atorvastatin) and relatively water-soluble (such as rosuvastatin) statins, depending on their polarity. This difference in inherent polarity may lead to variances in the availability of statins in different bones and individuals. Experiments have confirmed that only relatively fat-soluble statins enhance the expression of BMP2 and promote the osteogenic process [85]. This enhancement effect can be abolished by the addition of mevalonate, a downstream metabolite of HMG-CoA reductase, which confirms that the enhancement effect of these statins is facilitated by the inhibition of HMG-CoA reductase [86]. In vitro studies have also shown that statins promote osteoblast differentiation by stimulating the expression of BMP2, vascular endothelial growth factor (VEGF), Bgalp, bone sialoprotein (BSP), and promoting mineralization [87, 88]. Statins can also inhibit osteoclast production by downregulating the expression of RANKL and upregulating the expression of osteoprotegerin (OPG) [89]. These data suggest that the pharmacological effects of statins may not only be limited to lipid reduction, but may also be applied in bone protective applications and interventions. However, the use of statins should also be carefully evaluated, given their known side effects. To summarize, these studies provide a broadening understanding of OP and highlight the potential for novel therapeutic interventions in the near future.

### Cholesterol metabolism & bone neoplasms

Common malignant bone tumors include osteosarcoma, Ewing’s sarcoma, and chondrosarcoma. Giant cell tumors of the bone (giant cell tumors, GCTs) are an intermediate tumor with the possibility of malignant transformation. The incidence of primary bone tumors is low, but the degree of malignancy is high, and they experience high rates of recurrence and metastasis, which has a serious impact on the motor function of patients. At present, the specific pathogenesis of bone tumors is unclear, but it has been established that there are many factors affecting their occurrence, proliferation, migration, and prognosis. Although there is no direct experimental evidence to confirm the specific effect of cholesterol metabolism on bone tumors, a number of pathways related to bone tumor development have been shown to be regulated by cholesterol. Therefore, these studies suggest that there may be a direct relationship between cholesterol metabolism and the development and pathology of primary bone tumors, which may provide a new therapeutic avenue for clinical intervention.

#### Osteosarcoma

Osteosarcoma is a genetically heterogeneous malignant tumor that is common in children and adolescents. It is characterized by rapid growth and early metastasis [90]. At present, the pathogenesis of osteosarcoma is not clear, and the heterogeneity of osteosarcoma makes it impossible to target; treatment relies on resection and chemotherapy [91] with poor results. Therefore, the interaction between different osteosarcoma cells and the supporting matrix may provide new insights for developing novel therapies. The increase in serum cholesterol and the level of various pro-inflammatory factors (such as TNF-α) lead to an increase in many kinds of inflammatory factors. These inflammatory reactions are involved in bone destruction and exist in a variety of metabolic disorders, including T2DM [76]. Some experiments have confirmed that extracellular vesicles (EVs) secreted by malignant osteosarcoma cells are selectively integrated into the membrane-related forms of TGF-β. TGF-β can induce MSCs to produce inflammatory Interleukin-6 (IL-6), and IL-6 is known to increase cancer-promoting functions in MSCs. This is often accompanied by the activation of signal transducer and activator of transcription (STAT3) in tumors and the formation of lung metastases. Intravenous administration of the IL-6 receptor antibody, tocilizumab, eradicated the cancer-promoting effect of MSCs in the experimental group [92]. This suggests that hypercholesterolemia may also promote the occurrence and development of osteosarcoma by activating various inflammatory factors. However, because high cholesterol can also inhibit the TGF-β / Wnt pathway [54, 55], which is essential for normal osteogenesis, it may indicate that patients with hyperlipidemia may have a higher or lower risk of osteosarcoma, but both conjectures lack the support of experimental results and clinical statistics at this stage; therefore, new studies are urgently needed.

#### Chondrosarcoma

Chondrosarcoma is a malignant tumor that occurs in chondrocytes and usually affects young and middle-aged people. Chondrosarcoma is divided into primary and secondary chondrosarcomas. Secondary chondrosarcoma refers to the malignant transformation of benign cartilage lesions, such as endophytic chondroma and osteochondroma. Chondrosarcoma is a common malignant bone tumor and the second most common bone malignancy after osteosarcoma, accounting for 10–15% of all malignant bone tumors [93]. However, in malignant tumors of the pelvis, the incidence increases to more than 20%. In the new classification of bone tumors produced in 2013, chondrosarcomas are classified into grades I–III according to their degree of malignancy and cellular differentiation [94]. Chondrosarcoma is not sensitive to radiotherapy or chemotherapy and the main treatment for these cancers remains surgical resection. At present, surgical interventions for low-grade chondrosarcomas remain controversial, as some scholars think that it should be removed as a whole, while others think that intracapsular curettage combined with liquid nitrogen and electrocautery is sufficient [95]. No specific molecular mechanism has been described for chondrosarcoma and there is currently no adequate targeted therapy. Recent studies have shown that the BMP pathway is hyper-activated in chondrosarcoma, and the degree of activation is related to tumor grade. This activation results in increased osteogenesis and the degree of malignancy [96]. Therefore, inhibiting this pathway has become a potential therapeutic avenue [76]; thus blocking the expression of Runx2, ALPL, and COL2A1, may reduce the malignant progression of these tumors, suggesting that high-cholesterol diets or other similar methods may be one way to improve the physiological synthesis of cholesterol during chemotherapy and surgical interventions in these patients to improve their prognosis.

#### Ewing’s sarcoma

Ewing’s sarcoma (ES) is the third most common malignant bone tumor, and was first reported by James Ewing in 1921. It usually occurs in adolescents and children [97], and lesions are mostly found at the end of long bones and soft tissues. Ewing’s sarcoma is highly malignant and prone to metastasis, and recent advances in treatment have resulted in significant improvements in therapeutic success [98, 99]. However, the prognosis of patients with metastasis, local resection, and recurrence remains poor. Therefore, a more optimized treatment is needed. In-depth studies have found that the insulin-like growth factor-1 (IGF-1) / IGF-1 receptor (IGF-1R) system plays an important role in the occurrence and development of Ewing’s sarcoma [100, 101]. When the ligand binds to the outer subunit of IGF-1R, the conformation of the transmembrane β subunit changes, which leads to autophosphorylation of cytoplasmic tyrosine kinase. IGF-1R then phosphorylates cellular substrates, including insulin receptor substrates 1–4. Subsequent activation includes multiple signaling pathways such as the mitogen-activated protein kinase (MAPK), extracellular signal-regulated kinase (ERK), and phosphatidylinositol 3-kinase (PI3-K) / protein kinaseB (PKB)pathway. These pathways promote tumor cell resistance and reduce apoptosis while increasing transcription, metabolism, proliferation, and growth. Therefore, inhibiting the abnormal activation of IGF-1R may be a critical therapeutic intervention for these tumors and may provide new therapeutic ideas for the treatment of advanced patients. However, at present, this treatment induces drug resistance and exhibits high rates of recurrence [102, 103]. Cholesterol metabolism is closely related to IGF-1R expression. Mevalonate and its downstream products, the intermediate products of the cholesterol biosynthesis pathway, induce the expression of IGF-1/IGF-1R, which can significantly improve the anti-apoptosis capacity of tumor cells [104]. Therefore, patients with hypercholesterolemia may have a higher risk of ES and increased difficulty in treatment. Statins can significantly reduce the levels of serum cholesterol, thus reducing the expression of mevalonate and reducing the isoprenylation of various proteins related to cell movement [105]. Therefore, statins seem to be a reasonable treatment option and may inhibit the proliferation of these cancers. For example, simvastatin can inhibit the proliferation of colon and prostate cancer cells by reducing the expression of IGF-1R, inhibiting IGF-1-induced ERK/Akt activation, and selectively and persistently activate pro-apoptotic ERK signaling to induce apoptosis. The apoptosis-inducing effects of simvastatin could be completely avoided by mevalonate pretreatment and partially reversed by PD98059. Although the therapeutic effect of statins on Ewing’s sarcoma has not been reported in the literature, its potential in treating Ewing’s sarcoma should not be overlooked.

#### Giant cell bone tumors (GCT)

Giant cell bone tumors were first reported by Jaffe in 1940. This is a common primary bone tumor often observed in clinical practice and is usually identified in 20–50 year olds. These tumors are more common in female patients and most primary lesions are located in the epiphysis. The source of GCT is unclear, and some scholars speculate that it may originate from bone marrow mesenchymal tissue. GCT cells have strong invasive properties despite the fact that they are intermediate tumors and exert significant dissolution and destruction of the bone niche. Relatively few of them react to new bone production or self-healing, and some pass through the bone cortex to form soft tissue masses. At present, surgical treatment remains the first-line intervention for GCT, and focused curettage is the primary surgical method. The over-expression of RANKL is its characteristic pathogenesis, which leads to the activation of osteoclasts, explaining its high osteolytic activity. Therefore, for lesions with difficult curettage or high risks of postoperative recurrence, it is recommended that tumor edge resection is augmented by adding RANKL inhibitors to reduce the activation and development of osteoclasts and increase bone mineral density. Hypercholesterolemia activates the RANK/RANKL pathway, enhancing osteoclast differentiation and fusion while inhibiting osteoblast proliferation and differentiation, interrupting the normal balance between osteoclasts and osteoblasts, aggravating the destructive effect of GCT. Therefore, GCT patients who present with comorbidities such as hyperlipidemia and diabetes, or who are postmenopausal at onset may have a poorer prognosis than their more “normal” counterparts.

## Discussion and future prospects

Metabolic diseases of the bone have emerged as a particularly hot topic worldwide, and lipid metabolism crosstalk frequently overlaps with bone metabolism.

Clinical studies have shown that patients with osteoporosis experience some degree of lipid metabolism dysfunction. Studies have shown that obese patients have stronger osteoclast activity, more significant osteoclast biomarker activity, and more bone destruction products in their serum. In addition, obese patients experience an increase in body weight, which increases mechanical and skeletal stress, synovial membrane wear and tear, and dendritic cell activation, which results in significant changes in the chemokine profiles of this niche, facilitating changes in mesenchymal stem cell-mediated bone repair. Adipose cell-derived PPAR-γ induces adipocyte differentiation in MSCs and inhibits osteoblastic differentiation. PPAR-γ mediates its effects by interacting with the PPAR response elements, inducing changes in the osteoblastic transcription factors, Runx2 and Osx, inhibiting their downstream signaling and reducing osteoblast differentiation. Recent studies have shown that PPAR-γ-mediated induction of c-Fos upregulation is essential for osteoclast function [58]. This means that there may be an avenue for promoting bone regeneration by inhibiting certain components of lipid metabolism. Patients with diabetes mellitus treated with rosiglitazone often complain of increased bone fragility as an unwanted side effect.

Statin, a commonly used inhibitor of HMG-CoA, has been shown to exert both lipid-lowering and bone-protective effects. Statins were previously applied to lower cholesterol; however, more recent studies have shown that inhibiting HMG-CoA seems to promote the activity of specific transcription factors and induce the expression of critical osteoblast genes. Other studies have shown that statins inhibit function and survival via their interactions with lipids and cholesterol and alter osteoclast survival. Several studies have shown that statins play a positive role in reducing the risk of fracture by promoting bone production and inhibiting osteolytic metastasis. These osteoprotective effects are also dependent on cumulative dose and drug intensity, and the effect on bone metabolism was first identified by screening for BMP2 activators in mice. The protective effects on individual bones may be different because of their inherent polarity and bone bioavailability. In vitro studies have shown that statins promote osteoblast differentiation by stimulating the expression of BMP2, VEGF, osteocalcin, and BSP, and promoting mineralization. In addition, several clinical trials have shown that only lipophilic statins significantly enhance the expression of BMP2, thus promoting osteoblast differentiation. This stimulatory effect suggests that the osteoprotective effect of statins may be due to a combination of its lipid-lowering effect and the downstream implications of these outcomes. Statins induce osteogenesis and bone formation and activate the BMP2 promoter. This activation can be reversed by the addition of mevalonate, a downstream metabolite of HMG-CoA reductase, suggesting that this activation is the result of the inhibition of HMG-CoA reductase. Although statins have notable side effects in both the muscle and liver tissues, most studies have shown that these compounds achieve good results in clinical treatment and may also have some effect on the prevention and treatment of osteoporosis, especially in hyperlipidemia cases complicated with osteoporosis. In summary, these results suggest that statins may be a key strategy in treating bone loss-related diseases.

Lipids include fatty acids, cholesterol, and phospholipids. Fatty acids, especially unsaturated fatty acids, contain carbon-carbon double bonds, which are vulnerable to free radical attacks by circulating compounds such as ROS, resulting in electron gain and loss reactions and the production of stable structures such as malondialdehyde (MDA). This can be used to determine oxidative stress levels. Since oxidation is involved in bone inflammation, anti-oxidation drugs and oxidation detection could be a possible therapeutic target. Ferroptosis can be inhibited by iron detergents such as ferrostatin-1 (fer-1). However, these attacks reduce the stability and increase the permeability of membrane lipids, resulting in the loss of osmotic gradients and changes in membrane surface molecules, which are recognized by macrophages, mediating cell death using a mechanism similar to that described for apoptosis, causing osmotic death [41, 42]. These reaction products can also reduce the stability of unsaturated carbon-carbon bonds and increase the sensitivity to ferroptosis [42]. There are dense bone collar cells on the matrix surface, which form an important barrier between the bone matrix and the internal environment, preventing the destruction of the matrix components. Studies have shown that chronic inflammatory diseases of the bones and joints, such as osteoarthritis and lupus arthritis, experience different degrees of destruction of the bone collar cells, which results in the differential release of degraded collagen and fibrous matrix components that are recognized by the immune system, inducing the immune response and leading to the abnormal activation of osteoclasts, resulting in severe bone erosion and destruction. Several studies have shown that the death of bone collar cells is comprehensive and complex, including pyroptosis, autophagy, apoptosis, and ferroptosis. Although bone collar cells undergo apoptosis, there is significantly more ferroptosis in these cells, making it a growing field of interest in bone remodeling and regeneration. In addition to iron overload, a large amount of unsaturated lipids also exerts a ferroptotic effect. High lipid levels translate to high ROS and other radical levels, which activate NF-κB signaling pathways and induce inflammation [106]. Collectively, these results suggest that obese people are more likely to experience collar destruction resulting from fatty oxide exposure and ferroptosis. Therefore, preventing the death of bone collar cells by inhibiting ferroptosis, may become important when developing new osteological drugs. Other agents such as deferoxamine (DFO) can also inhibit ferroptosis. Recent studies have shown that FSP-1 is often recruited during these inhibitory effects [42]. Thus, anti-ferroptosis agents may become the next major drug target for developing treatments for bone loss.

Economic development and social progress resulted in obesity and hyperlipidemia gradually becoming important social issues, as they are often the primary causes of a variety of diseases. Obesity is no longer the sole burden of obese patients but has gradually become the burden of the whole society [107]. Fatty acids and cholesterol are could be stressful environments, and the cytokines produced by fat cells exert numerous inflammatory effects that often exacerbate other underlying conditions [106]. In summary, previous studies have investigated the potential relationship between lipid or cholesterol metabolism and bone metabolism at the cellular level. However, an overview of the balance between lipids and bone has not yet been elucidated. In this review, the interplay of lipids and bones in physiological or pathophysiological stages was summarized at the cell interaction and molecular level. However, the subcellular signal transduction of how lipids or cholesterol influeence bone cells remains to be investigated, especially the newly discovered cell death processes such as ferroptosis and hedgehog signaling. Studies have shown that cholesterol may function as an endogenous ligand of hedgehog signaling, which alters the development and differentiation of bone cells. The detailed molecular pathway of how lipid-and cholesterol-derived ROS make sense has not been elucidated. In addition, a multi-center large-sample random control test should be conducted to identify the first-line drug administration of bone illness with hyperlipidemia.

## Conclusion

High cholesterol is related to high morbidity in bone destruction diseases, increased bone loss, and overall reductions in health, all of which have a significant economic impact. The findings of recent studies discussed in this review indicated the importance of decreasing fatty acid and cholesterol levels in patients to protect their bone health. Lipid and cholesterol metabolism-related drugs should be used in patients with or without future risk of obesity. The oxidation relative substrate detection method may be utilized in orthopedic patients with a high BMI (Table 1).

**Table 1:** Factors and cytokines associated with bone mass regulation

Factor	Derivation	Effect	Reference
Notch	osteoblasts osteocytes	Bidirectional bone formation and absorption	[23–25]
TNF-α IL-1β IL-6	Dendritic cell Macrophage	Increases bone absorption	[11]
RANKL	osteoblasts osteocytes Dendritic cell Adipocyte Fibroblast	Osteoclast differentiation and development Essential for bone absorption	[12]
OPG	osteoblasts osteocytes	RANKL inhibition Bone protection	[10]
PPAR-γ	Adipocyte	Inhibits bone formation Increases bone absorption	[18]
Leptin	Adipocyte	Increased osteoblast differentiation of MSCs Promotes bone formation Inhibits adipocyte accumulation	[20]
Adiponectin	Adipocyte	Increases bone formation Inhibits adipocyte accumulation	[20]

## Data Availability

Not applicable.

## Abbreviations

HMG-CoA: 3-hydroxy-3-methylglutaryl-CoA
OB: Osteoblasts
OC: Osteoclasts
HSCs: Hematopoietic stem cells
MSCs: Mesenchymal stem cells
M-CSF: Macrophage colony-stimulating factor
RA: Rheumatoid arthritis
HCD: High cholesterol diets
TRAP: Tartrate resistant acid phosphatase
BMP: Bone morphogenetic protein
iDC: Immature dendritic cells
LPA: Lysophosphatidic acid
PTH: Parathyroid hormone
PUFA-OO: Ploy-unsaturated fatty acid radicals
TNF: Tumor necrosis factor
TRAF6: TNF-receptor associated factor 6
MMPs: Matrix metalloproteinases
OP: Osteoporosis
TG: Triglyceride
TC: Total cholesterol
LDL-C: Low density lipoprotein cholesterol
ALP: Alkaline phosphatase
COL2A1: Collagen type 1
BMP2: Bone morphogenetic protein
Runx2: Dwarf related transcription factor 2
LDLR: Lipoprotein receptor negative
ApoA: Apolipoprotein A
ApoB: Apolipoprotein B
VEGF: Vascular endothelial growth factor
BSP: Bgalp and bone sialoprotein
OPG: Osteoprotegerin
IGF-1: Insulin-like growth factor-1
IGF-1R: IGF-1 receptor
MAPK: Mitogen-activated protein kinase
ERK: Extracellular signal-regulated kinase
PI3-K: Phosphatidylinositol 3-kinase
GCT: Giant cell bone tumors

## References

[1] Seeman E (2002). Pathogenesis of bone fragility in women and men. Lancet..

[2] Tian L, Yu X (2015). Lipid metabolism disorders and bone dysfunction--interrelated and mutually regulated (review). Mol Med Rep.

[3] Udagawa N, Koide M, Nakamura M, Nakamichi Y, Yamashita T, Uehara S (2021). Osteoclasts differentiation by RANKL and OPG signaling pathways. J Bone Miner Metab.

[4] Ma X, Yu J (2020). Role of the bone microenvironment in bone metastasis of malignant tumors - therapeutic implications. Cell Oncol (Dordr).

[5] Deligiorgi MV, Panayiotidis MI, Griniatsos J, Trafalis DT (2020). Harnessing the versatile role of OPG in bone oncology: counterbalancing RANKL and TRAIL signaling and beyond. Clin Exp Metastasis.

[6] Riquelme MA, Cardenas ER, Xu H, Jiang JX. The role of connexin channels in the response of mechanical loading and unloading of bone. Int J Mol Sci. 2020;21(3). 10.3390/ijms21031146. 10.3390/ijms21031146 PMC7038207 32050469

[7] Kurenkova AD, Medvedeva EV, Newton PT, Chagin AS (2020). Niches for skeletal stem cells of mesenchymal origin. Front Cell Dev Biol.

[8] Ma QL, Fang L, Jiang N, Zhang L, Wang Y, Zhang YM (2018). Bone mesenchymal stem cell secretion of sRANKL/OPG/M-CSF in response to macrophage-mediated inflammatory response influences osteogenesis on nanostructured Ti surfaces. Biomaterials..

[9] Taniguchi R, Inoue A, Sayama M, Uwamizu A, Yamashita K, Hirata K (2017). Structural insights into ligand recognition by the lysophosphatidic acid receptor LPA(6). Nature..

[10] Baud'huin M, Lamoureux F, Duplomb L, Rédini F, Heymann D (2007). RANKL, RANK, osteoprotegerin: key partners of osteoimmunology and vascular diseases. Cell Mol Life Sci.

[11] Shoji-Matsunaga A, Ono T, Hayashi M, Takayanagi H, Moriyama K, Nakashima T (2017). Osteocytes regulation of orthodontic force-mediated tooth movement via RANKL expression. Sci Rep.

[12] Honma M, Ikebuchi Y, Suzuki H (2021). RANKL as a key figure in bridging between the bone and immune system: its physiological functions and potential as a pharmacological target. Pharmacol Ther.

[13] Mulholland BS, Forwood MR, Morrison NA (2019). Monocyte chemoattractant protein-1 (MCP-1/CCL2) drives activation of bone remodelling and skeletal metastasis. Curr Osteoporos Rep.

[14] Park JH, Lee NK, Lee SY (2017). Current understanding of RANK signaling in osteoclasts differentiation and maturation. Mol Cell.

[15] Papachristou NI, Blair HC, Kalyvioti ES, Syggelos SA, Karavia EA, Kontogeorgakos V (2018). Western-type diet differentially modulates osteoblasts, osteoclasts, and lipoblast differentiation and activation in a background of APOE deficiency. Lab Investig.

[16] Lin YH, Kang L, Feng WH, Cheng TL, Tsai WC, Huang HT, et al. Effects of lipids and lipoproteins on mesenchymal stem cells used in cardiac tissue regeneration. Int J Mol Sci. 2020;21(13). 10.3390/ijms21134770. 10.3390/ijms21134770 PMC7369828 32635662

[17] Hurwitz SN, Jung SK, Kurre P (2020). Hematopoietic stem and progenitor cell signaling in the niche. Leukemia..

[18] Li X, Ning L, Ma J, Xie Z, Zhao X, Wang G (2019). The PPAR-γ antagonist T007 inhibits RANKL-induced osteoclastsogenesis and counteracts OVX-induced bone loss in mice. Cell Commun Signal.

[19] Guo Y, Huo J, Wu D, Hao H, Ji X, Zhao E (2020). Simvastatin inhibits the adipogenesis of bone marrow-derived mesenchymal stem cells through the downregulation of chemerin/CMKLR1 signaling. Int J Mol Med.

[20] Pawlak D, Domaniewski T, Znorko B, Oksztulska-Kolanek E, Lipowicz P, Doroszko M (2017). The impact of peripheral serotonin on leptin-brain serotonin axis, bone metabolism and strength in growing rats with experimental chronic kidney disease. Bone..

[21] Perakakis N, Farr OM, Mantzoros CS (2021). Leptin in leanness and obesity: JACC state-of-the-art review. J Am Coll Cardiol.

[22] Wang P, Loh KH, Wu M, Morgan DA, Schneeberger M, Yu X (2020). A leptin-BDNF pathway regulating sympathetic innervation of adipose tissue. Nature..

[23] Tikhonova AN, Dolgalev I, Hu H, Sivaraj KK, Hoxha E, Cuesta-Domínguez Á (2019). The bone marrow microenvironment at single-cell resolution. Nature..

[24] Colombo M, Platonova N, Giannandrea D, Palano MT, Basile A, Chiaramonte R (2019). Re-establishing apoptosis competence in bone associated cancers via communicative reprogramming induced through notch signaling inhibition. Front Pharmacol.

[25] Guo Z, Ohlstein B. Stem cell regulation. Bidirectional notch signaling regulates Drosophila intestinal stem cell multipotency. Science. 2015;350(6263). 10.1126/science.aab0988. 10.1126/science.aab0988 PMC5431284 26586765

[26] Shilo BZ, Sprinzak D (2017). The lipid-binding side of notch ligands. EMBO J.

[27] Shen G, Ren H, Shang Q, Zhao W, Zhang Z, Yu X (2020). Foxf1 knockdown promotes BMSC osteogenesis in part by activating the Wnt/β-catenin signalling pathway and prevents ovariectomy-induced bone loss. EBioMedicine..

[28] Yang T, Sun W, Duan YH, Sun YB, Ren YM, Hou WY (2020). Vitamin D3 protects articular cartilage by inhibiting the Wnt/β-catenin signaling pathway. Exp Ther Med.

[29] Manivel VA, Sohrabian A, Wick MC, Mullazehi M, Håkansson LD, Rönnelid J (2015). Anti-type II collagen immune complex-induced granulocyte reactivity is associated with joint erosions in RA patients with anti-collagen antibodies. Arthritis Res Ther.

[30] Peretz A, Body JJ, Dumon JC, Rozenberg S, Hotimski A, Praet JP (1996). Cyclical pamidronate infusions in postmenopausal osteoporosis. Maturitas..

[31] Ambele MA, Dhanraj P, Giles R, Pepper MS. Adipogenesis: a complex interplay of multiple molecular determinants and pathways. Int J Mol Sci. 2020;21(12). 10.3390/ijms21124283. 10.3390/ijms21124283 PMC7349855 32560163

[32] Hou C, Luan L, Ren C (2018). Oxidized low-density lipoprotein promotes osteoclasts differentiation from CD68 positive mononuclear cells by regulating HMGB1 release. Biochem Biophys Res Commun.

[33] Yin W, Li Z, Zhang W. Modulation of bone and marrow niche by cholesterol. Nutrients. 2019;11(6). 10.3390/nu11061394. 10.3390/nu11061394 PMC6628005 31234305

[34] Luegmayr E, Glantschnig H, Wesolowski GA, Gentile MA, Fisher JE, Rodan GA (2004). Osteoclasts formation, survival and morphology are highly dependent on exogenous cholesterol/lipoproteins. Cell Death Differ.

[35] Rogers MJ, Mönkkönen J, Munoz MA (2020). Molecular mechanisms of action of bisphosphonates and new insights into their effects outside the skeleton. Bone..

[36] Caetano-Lopes J, Canhão H, Fonseca JE (2009). Osteoimmunology--the hidden immune regulation of bone. Autoimmun Rev.

[37] Portal-Núñez S, Mediero A, Esbrit P, Sánchez-Pernaute O, Largo R, Herrero-Beaumont G (2017). Unexpected bone formation produced by RANKL blockade. Trends Endocrinol Metab.

[38] Pal China S, Sanyal S, Chattopadhyay N (2018). Adiponectin signaling and its role in bone metabolism. Cytokine..

[39] Tomofuji T, Ekuni D, Azuma T, Irie K, Endo Y, Yamamoto T (2012). Supplementation of broccoli or Bifidobacterium longum-fermented broccoli suppresses serum lipid peroxidation and osteoclasts differentiation on alveolar bone surface in rats fed a high-cholesterol diet. Nutr Res.

[40] Sanbe T, Tomofuji T, Ekuni D, Azuma T, Irie K, Tamaki N (2009). Vitamin C intake inhibits serum lipid peroxidation and osteoclasts differentiation on alveolar bone in rats fed on a high-cholesterol diet. Arch Oral Biol.

[41] Riegman M, Sagie L, Galed C, Levin T, Steinberg N, Dixon SJ (2020). Ferroptosis occurs through an osmotic mechanism and propagates independently of cell rupture. Nat Cell Biol.

[42] Zou Y, Henry WS, Ricq EL, Graham ET, Phadnis VV, Maretich P (2020). Plasticity of ether lipids promotes ferroptosis susceptibility and evasion. Nature..

[43] Kosmin M, Padhani AR, Gogbashian A, Woolf D, Ah-See ML, Ostler P (2020). Comparison of whole-body MRI, CT, and bone scintigraphy for response evaluation of cancer therapeutics in metastatic breast cancer to bone. Radiology..

[44] Tousoulis D, Papageorgiou N, Androulakis E, Siasos G, Latsios G, Tentolouris K (2013). Diabetes mellitus-associated vascular impairment: novel circulating biomarkers and therapeutic approaches. J Am Coll Cardiol.

[45] Bersuker K, Hendricks JM, Li Z, Magtanong L, Ford B, Tang PH (2019). The CoQ oxidoreductase FSP1 acts parallel to GPX4 to inhibit ferroptosis. Nature..

[46] Mazière C, Salle V, Gomila C, Mazière JC (2013). Oxidized low density lipoprotein enhanced RANKL expression in human osteoblasts-like cells. Involvement of ERK, NFkappaB and NFAT. Biochim Biophys Acta.

[47] Ohgi K, Kajiya H, Goto TK, Okamoto F, Yoshinaga Y, Okabe K (2018). Toll-like receptor 2 activation primes and upregulates osteoclastsogenesis via lox-1. Lipids Health Dis.

[48] Ascone G, Di Ceglie I, Walgreen B, Sloetjes AW, Lindhout E, Bot I (2020). High LDL levels lessen bone destruction during antigen-induced arthritis by inhibiting osteoclasts formation and function. Bone..

[49] Mazière C, Louvet L, Gomila C, Kamel S, Massy Z, Mazière JC (2009). Oxidized low density lipoprotein decreases Rankl-induced differentiation of osteoclasts by inhibition of Rankl signaling. J Cell Physiol.

[50] Richard E, Marian S. Prevention and management of osteoporosis. Medicine. 2021;49(9):572–577. 10.1016/j.mpmed.2021.06.010.

[51] Barengolts EI, Berman M, Kukreja SC, Kouznetsova T, Lin C, Chomka EV (1998). Osteoporosis and coronary atherosclerosis in asymptomatic postmenopausal women. Calcif Tissue Int.

[52] Yao W, Cheng Z, Busse C, Pham A, Nakamura MC, Lane NE (2008). Glucocorticoid excess in mice results in early activation of osteoclastsogenesis and adipogenesis and prolonged suppression of osteogenesis: a longitudinal study of gene expression in bone tissue from glucocorticoid-treated mice. Arthritis Rheum.

[53] You L, Sheng ZY, Tang CL, Chen L, Pan L, Chen JY (2011). High cholesterol diet increases osteoporosis risk via inhibiting bone formation in rats. Acta Pharmacol Sin.

[54] Hill TP, Später D, Taketo MM, Birchmeier W, Hartmann C (2005). Canonical Wnt/beta-catenin signaling prevents osteoblasts from differentiating into chondrocytes. Dev Cell.

[55] Glass DA, Bialek P, Ahn JD, Starbuck M, Patel MS, Clevers H (2005). Canonical Wnt signaling in differentiated osteoblasts controls osteoclasts differentiation. Dev Cell.

[56] Parhami F, Tintut Y, Beamer WG, Gharavi N, Goodman W, Demer LL (2001). Atherogenic high-fat diet reduces bone mineralization in mice. J Bone Miner Res.

[57] Pelton K, Krieder J, Joiner D, Freeman MR, Goldstein SA, Solomon KR (2012). Hypercholesterolemia promotes an osteoporotic phenotype. Am J Pathol.

[58] Sanbe T, Tomofuji T, Ekuni D, Azuma T, Tamaki N, Yamamoto T (2007). Oral administration of vitamin C prevents alveolar bone resorption induced by high dietary cholesterol in rats. J Periodontol.

[59] Ishikawa S, Iwasaki K, Komaki M, Ishikawa I (2004). Role of ascorbic acid in periodontal ligament cell differentiation. J Periodontol.

[60] Bergstrom JD, Bostedor RG, Masarachia PJ, Reszka AA, Rodan G (2000). Alendronate is a specific, nanomolar inhibitor of farnesyl diphosphate synthase. Arch Biochem Biophys.

[61] Okayasu M, Nakayachi M, Hayashida C, Ito J, Kaneda T, Masuhara M (2012). Low-density lipoprotein receptor deficiency causes impaired osteoclastsogenesis and increased bone mass in mice because of defect in osteoclastsic cell-cell fusion. J Biol Chem.

[62] Widlansky ME, Gokce N, Keaney JF, Vita JA (2003). The clinical implications of endothelial dysfunction. J Am Coll Cardiol.

[63] Wang GJ, Moga DB, Richemer WG, Sweet DE, Reger SI, Thompson RC (1978). Cortisone induced bone changes and its response to lipid clearing agents. Clin Orthop Relat Res.

[64] Kitajima M, Shigematsu M, Ogawa K, Sugihara H, Hotokebuchi T (2007). Effects of glucocorticoid on adipocyte size in human bone marrow. Med Mol Morphol.

[65] Miyanishi K, Yamamoto T, Irisa T, Yamashita A, Jingushi S, Noguchi Y (2002). Bone marrow fat cell enlargement and a rise in intraosseous pressure in steroid-treated rabbits with osteonecrosis. Bone..

[66] Zhou Q, Li Q, Yang L, Liu F (2000). Changes of blood vessels in glucocorticoid-induced avascular necrosis of femoral head in rabbits. Zhonghua Wai Ke Za Zhi.

[67] Broulik PD, Kapitola J (1993). Interrelations between body weight, cigarette smoking and spine mineral density in osteoporotic Czech women. Endocr Regul.

[68] Tang YJ, Sheu WH, Liu PH, Lee WJ, Chen YT (2007). Positive associations of bone mineral density with body mass index, physical activity, and blood triglyceride level in men over 70 years old: a TCVGHAGE study. J Bone Miner Metab.

[69] Parhami F, Mody N, Gharavi N, Ballard AJ, Tintut Y, Demer LL (2002). Role of the cholesterol biosynthetic pathway in osteoblastsic differentiation of marrow stromal cells. J Bone Miner Res.

[70] Samelson EJ, Cupples LA, Hannan MT, Wilson PW, Williams SA, Vaccarino V (2004). Long-term effects of serum cholesterol on bone mineral density in women and men: the Framingham osteoporosis study. Bone..

[71] Tankó LB, Bagger YZ, Nielsen SB, Christiansen C (2003). Does serum cholesterol contribute to vertebral bone loss in postmenopausal women?. Bone..

[72] Li K, Xiu C, Zhou Q, Ni L, Du J, Gong T (2019). A dual role of cholesterol in osteogenic differentiation of bone marrow stromal cells. J Cell Physiol.

[73] Yamaguchi T, Sugimoto T, Yano S, Yamauchi M, Sowa H, Chen Q (2002). Plasma lipids and osteoporosis in postmenopausal women. Endocr J.

[74] Chen YY, Wang WW, Yang L, Chen WW, Zhang HX (2018). Association between lipid profiles and osteoporosis in postmenopausal women: a meta-analysis. Eur Rev Med Pharmacol Sci.

[75] Edwards CJ, Hart DJ, Spector TD (2000). Oral statins and increased bone-mineral density in postmenopausal women. Lancet..

[76] Chen Z, Zhao GH, Zhang YK, Shen GS, Xu YJ, Xu NW (2017). Research on the correlation of diabetes mellitus complicated with osteoporosis with lipid metabolism, adipokines and inflammatory factors and its regression analysis. Eur Rev Med Pharmacol Sci.

[77] Sivas F, Alemdaroğlu E, Elverici E, Kuluğ T, Ozoran K (2009). Serum lipid profile: its relationship with osteoporotic vertebrae fractures and bone mineral density in Turkish postmenopausal women. Rheumatol Int.

[78] Dennison EM, Syddall HE, Aihie Sayer A, Martin HJ, Cooper C (2007). Lipid profile, obesity and bone mineral density: the Hertfordshire cohort study. Qjm..

[79] Mandal CC (2015). High cholesterol deteriorates bone health: new insights into molecular mechanisms. Front Endocrinol (Lausanne).

[80] An T, Hao J, Sun S, Li R, Yang M, Cheng G (2017). Efficacy of statins for osteoporosis: a systematic review and meta-analysis. Osteoporos Int.

[81] Yamauchi M, Yamaguchi T, Nawata K, Tanaka K, Takaoka S, Sugimoto T (2015). Increased low-density lipoprotein cholesterol level is associated with non-vertebral fractures in postmenopausal women. Endocrine..

[82] Makovey J, Chen JS, Hayward C, Williams FM, Sambrook PN (2009). Association between serum cholesterol and bone mineral density. Bone..

[83] Lin TK, Chou P, Lin CH, Hung YJ, Jong GP (2018). Long-term effect of statins on the risk of new-onset osteoporosis: a nationwide population-based cohort study. PLoS One.

[84] Mundy G, Garrett R, Harris S, Chan J, Chen D, Rossini G (1999). Stimulation of bone formation in vitro and in rodents by statins. Science..

[85] Oryan A, Kamali A, Moshiri A (2015). Potential mechanisms and applications of statins on osteogenesis: current modalities, conflicts and future directions. J Control Release.

[86] Horiuchi N, Maeda T (2006). Statins and bone metabolism. Oral Dis.

[87] Arpornmaeklong P, Pripatnanont P, Chookiatsiri C, Tangtrakulwanich B (2017). Effects of titanium surface microtopography and simvastatin on growth and osteogenic differentiation of human mesenchymal stem cells in estrogen-deprived cell culture. Int J Oral Maxillofac Implants.

[88] Lee H, Lee H, Na CB, Park JB (2018). Effects of simvastatin on the viability and secretion of vascular endothelial growth factor of cell spheroids cultured in growth media. Implant Dent.

[89] Tsubaki M, Satou T, Itoh T, Imano M, Yanae M, Kato C (2012). Bisphosphonate- and statin-induced enhancement of OPG expression and inhibition of CD9, M-CSF, and RANKL expressions via inhibition of the Ras/MEK/ERK pathway and activation of p38MAPK in mouse bone marrow stromal cell line ST2. Mol Cell Endocrinol.

[90] Simpson S, Dunning MD, de Brot S, Grau-Roma L, Mongan NP, Rutland CS (2017). Comparative review of human and canine osteosarcoma: morphology, epidemiology, prognosis, treatment and genetics. Acta Vet Scand.

[91] Mirabello L, Troisi RJ, Savage SA (2009). Osteosarcoma incidence and survival rates from 1973 to 2004: data from the surveillance, epidemiology, and end results program. Cancer..

[92] Baglio SR, Lagerweij T, Pérez-Lanzón M, Ho XD, Léveillé N, Melo SA (2017). Blocking tumor-educated MSC paracrine activity halts osteosarcoma progression. Clin Cancer Res.

[93] Samuel AM, Costa J, Lindskog DM (2014). Genetic alterations in chondrosarcomas - keys to targeted therapies?. Cell Oncol (Dordr).

[94] Chang L, Shrestha S, LaChaud G, Scott MA, James AW (2015). Review of microRNA in osteosarcoma and chondrosarcoma. Med Oncol.

[95] Veth R, Schreuder B, van Beem H, Pruszczynski M, de Rooy J (2005). Cryosurgery in aggressive, benign, and low-grade malignant bone tumours. Lancet Oncol.

[96] Boeuf S, Bovée JV, Lehner B, van den Akker B, van Ruler M, Cleton-Jansen AM (2012). BMP and TGFbeta pathways in human central chondrosarcoma: enhanced endoglin and Smad 1 signaling in high grade tumors. BMC Cancer.

[97] Burningham Z, Hashibe M, Spector L, Schiffman JD (2012). The epidemiology of sarcoma. Clin Sarcoma Res.

[98] van Maldegem AM, Bovée JV, Peterse EF, Hogendoorn PC, Gelderblom H (2016). Ewing sarcoma: the clinical relevance of the insulin-like growth factor 1 and the poly-ADP-ribose-polymerase pathway. Eur J Cancer.

[99] Theisen ER, Pishas KI, Saund RS, Lessnick SL (2016). Therapeutic opportunities in Ewing sarcoma: EWS-FLI inhibition via LSD1 targeting. Oncotarget..

[100] Vezzali R, Weise SC, Hellbach N, Machado V, Heidrich S, Vogel T (2016). The FOXG1/FOXO/SMAD network balances proliferation and differentiation of cortical progenitors and activates Kcnh3 expression in mature neurons. Oncotarget..

[101] Wang C, Liu W, Zhang X, Wang Y, Liu H, Li H (2017). MEK/ERK signaling is involved in the role of VEGF and IGF1 in cardiomyocyte differentiation of mouse adipose tissue-derived stromal cells. Int J Cardiol.

[102] Huang L, Nakai Y, Kuwahara I, Matsumoto K (2012). PRAS40 is a functionally critical target for EWS repression in Ewing sarcoma. Cancer Res.

[103] Sasaki M, Hasegawa T, Hongo H, Yamamoto T, Amizuka N (2013). Bone histology after intermittent PTH treatment - animal models. Clin Calcium.

[104] Sharon C, Baranwal S, Patel NJ, Rodriguez-Agudo D, Pandak WM, Majumdar N, Krystal G, Patel B (2015). Inhibition of insulin-like growth factor receptor/AKT/mammalian target of rapamycin axis targets colorectal cancer stem cells by attenuating mevalonate-isoprenoid pathway in vitro and in vivo. Oncotarget..

[105] Sanyour HJ, Li N, Rickel AP, Torres HM, Anderson RH, Miles MR (2020). Statin-mediated cholesterol depletion exerts coordinated effects on the alterations in rat vascular smooth muscle cell biomechanics and migration. J Physiol.

[106] Hashimoto K, Akagi M (2020). The role of oxidation of low-density lipids in pathogenesis of osteoarthritis: a narrative review. J Int Med Res.

[107] Chen XW, Ding G, Xu L, Li P. A glimpse at the metabolic research in China. Cell Metab. 10.1016/j.cmet.2021.09.014. undefined. 10.1016/j.cmet.2021.09.014 34619075

